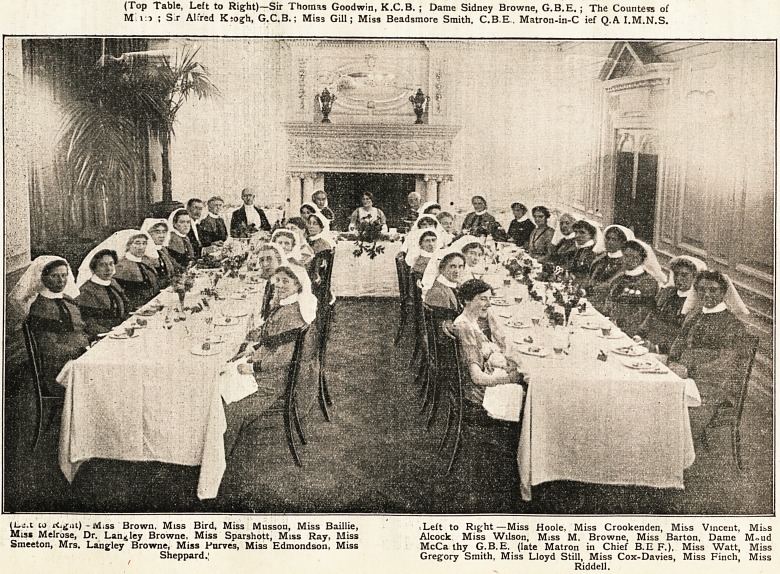# Dinner to Dame Sidney Browne, Matron-In-Chief, T.F.N.S.

**Published:** 1919-12-13

**Authors:** 


### DINNER TO DAME SIDNEY BROWNE.

### Matron-in-Chief, T.F.N.S.

An event of historical interest took place on Wednesday evening, December 3, at the Grosvenor Hotel, in the shape of an informal and complimentary dinner to Dame Sidney Browne, G.B.E., R.R.C., given by the twenty-five Principal Matrons of the T.F.N.S. on the eve of her retirement from active service. They were :

London.—Miss Cox-Davies, R.R.C.; Miss Finch, R.R.C.; Miss Barton, R.R.C.; Miss Ray, R.R.C.; Miss Lloyd-Still, C.B.E., R.R.C., and Miss Riddell, R.R.C.

Southern.—Miss Musson, R.R.C.; Miss Baillie, R.R.C.; Miss Watt, R.R.C.; Miss Smale, R.R.C.; and Miss Alcock, R.R.C.

Eastern.—Miss Crockenden, R.R.C., and Miss Bird, R.R.C.

Western.—Miss Purves, R.R.C.; Miss Sparshott, C.B.E., R.R.C.; and Miss Wilson, R.R.C.

Northern.—Miss Brown, R.R.C.; Miss Innes, R.R.C.; Miss Smeeton, R.R.C.; Miss Sheppard, R.R.C.; and Miss Vincent, R.R.C.

Scottish.—Miss Edmondson, R.R.C.; Miss Gill, R.R.C.; Miss Gregory Smith, R.R.C.; and Miss Melrose, R.R.C.

The Countess of Minto presided, and Sir Alfred Keogh, G.C.B., G.C.V.O., C.H., Sir Thomas Goodwin, K.C.B., D.G.A.M.S., Dr. Langley Browne and Mrs. Browne (brother and sister-in-law), Dame Maude McCarthy, G.B.E., R.R.C., Miss Beadsmore Smith, C.B.E., R.R.C., Miss Brown, and Miss Hoole, were present, making thirty-three in all. Miss Innes and Miss Smale were unfortunately absent owing to illness. The tables were decorated with scarlet geraniums, smilax, and white heather, and the menu bore the badge of the Service in colours. Selections of music were played during the dinner, at the conclusion of which there was a general exchange of autographs on the menu cards. The toast of "The King" was proposed by Lady Minto, and complied with in true military style and spirit. This was followed by Sir Thomas Goodwin's toast to Queen Alexandra, and to the Services. He added "it was impossible to make a speech, as no oratory could express the feelings all of them felt."

**Miss Cox-Davies, R.R.C.,** rose to propose the health of "Our Matron-in-Chief." In delightful allusion and tribute Miss Cox-Davies voiced the thoughts and the feelings of all present. She prefaced her remarks by emphasising the honour and privilege it was to depict the long years of service, of splendid work in many countries, of which Dame Sidney could be justly proud. It was not often the privilege came to hold a celebrity at one's mercy such as the present occasion afforded, but dropping all formality the speaker said she would in more friendly and homely fashion describe briefly three pictures that were outstanding in her memory of "our Dame."

### South Africa.

Episode 1 went back many years to the South African War, at the time of a severe typhoid epidemic in a certain hospital, when the sisters took twenty-four hours of duty. It was a time of dust storms, or, as they were termed, "scratchy atmosphere," when home-sickness and longing for England and civilisation attacked everyone with intensity. In the superintending sister of that particular hospital there was something soul-arresting. Her firm attitude, her cheery voice, and great friendliness never failed to cure the malaise of soul. She held out the strong hand of friendship to all, filled one with a cheery sense of optimism, and was always level-headed and sane in a simple, unconscious way.

### Coronation Day.

Episode 2 occurred many years later, on the Coronation Day of King George, and incidentally the birthday of the T.F.N.S. Invitations were sent to this young Force to make their bow to their King. There existed then no regulations regarding uniform, but in her customary manner the matron-in-chief dealt with the difficulty. The rendezvous was at Middlesex Hospital, and no service ever mustered a set of such extraordinarily garbed people. But with serene countenance and firm, proud step, "Our Matron-in-Chief" marched at the head of her force through the streets, up Constitution Hill, heedless, regardless of the little boys who ran after them, conscious only of the thought, though nobody knew it, of the great and powerful force it would eventually become.

### The Triumphal March.

Many years later, again, Episode 3, July 5, 1919, the Triumphal March through the London streets, when, with the troops, and this time no nonsense about uniform, the Nursing Services marched past their King and Queen, proud to show their war decorations, prouder still to see the commendation in the eye of their Matron-in-Chief, and again still prouder to see her receiving the Royal congratulations from their Majesties. The Service had made good. Our Matron-in-Chief's wonderful work had fought for a high standard and attained it through her wonderful gift of sympathy, her remarkable courtesy, which had endeared her to every member in every unit. She possessed the woman's soldier spirit; she was ever mindful of the well-being of the sick. "She is a great lady whom we all love, and I ask you to drink to her health." Amid great enthusiasm and cheers the health of Dame Sidney Browne was drunk, the guests lustily singing "For she's a jolly good fellow." On rising to reply Dame Sidney was considerably touched by the ovation and tribute she had received.

### Dame Sidney Browne's Reply.

Vice-President, Ladies and Gentlemen,—I cannot express what I feel about your great kindness in inviting me to this dinner, and for the delightful greeting from all the Principal Matrons expressed by Miss Cox-Davies. I shall shortly be leaving this Service, which we have all built up together. It was started by Miss Haldane and presided over by Queen Alexandra. I shall never forget this day or the splendid work you have done.' I consider the Principal Matrons to be the backbone of the Service, and there is, and always has been, a spirit of good fellowship and comradeship between us. You have always been most loyal. We have worked together on the happiest terms, and I shall be very sorry indeed to say goodbye to you. I am delighted to see the late Director-General, Sir Alfred Keogh, here, for if it had not been for the help he gave in the long years of preparation before the war, the Service could not have done the great work it has done. He was always ready to help and advise us, and though his own medical work was so extremely difficult, as the medical work always will be in war time, when it is impossible to foresee what may happen, and prepara-

Continued on pages 243 and 244.

### Dinner to Dame Sidney Browne: December 3, 1919.

(*See pages 242 and 244.*)

**Dinner to Dame Sidney Browne**—(*continued*).

tions have to be made in the dark, yet in the middle of his work he was always ready to advise and help the Nursing Services, and as long as he was satisfied that the three Matrons-in-Chief were providing sufficiently for the nursing of the wounded, he trusted them to organise and direct their own Services, and it was a great pleasure and honour to serve under him. The other two Matrons-in-Chief with whom I have served were most delightful comrades. We worked together very happily with no friction whatever, and they helped and encouraged the members of our Service just as much as they did their own, and all recommendations for promotion and honours were equally and fairly distributed.

We owe a great debt of gratitude to the Principal Matrons. They have never failed us, and though I realise I have made their work very difficult at times when I have asked for thousands of nurses from the Territorial Hospitals at home to serve overseas, they have always carried out our wishes, and again and again loyally sent us their best nurses, and have had to fill their places with less experienced newly enrolled members. On one occasion when we had just taken a large number of nurses away for overseas, 100 more were required at the shortest notice, and the Commanding Officers and Matrons said it was almost impossible to spare them, but the Director-General said it was necessary for them to go. Nurses or substitutes for nurses could be obtained easier in England than in Mesopotamia or Salonica, and they were immediately forthcoming. This alone, apart from the excellent work carried out in organising the nursing arrangements at home, was one of the greatest helps in time of war, and we cannot be too grateful to the Principal Matrons for all they have done. It is delightful to have the Director-General here, too, as he has every sympathy with the Service. I must say a word of thanks to Lady Minto. She has been the Vice-President of our Council since the formation of the Service in 1908, and has cheered and helped us by her sympathy and encouragement all through the war, and though she has suffered herself so much through the war, yet she has brought happiness and brightness to us even in those dark days, and we owe much to her unselfish inspiration and encouragement.

**Lady Minto** then recalled the delightful association she had had with Dame Sidney Browne, and the work of the Principal Matrons who were the backbone of the Service, and to whom a great debt of gratitude was owed. She was followed by Sir Alfred Keogh, who responded to the toast of the visitors, proposed by Miss Sparshott, and told the story of the Nursing Service established after the Crimean War. He paid a glowing tribute to Dame Sidney's work during the South African War, saying that whatever credit there was for the efficiency of the Nursing Service, he could without hesitation affirm it was due to her characteristic and extraordinary sympathy with her nurses. Deeply attached to her as a friend, he was proud to be with them in giving her honour, and as her chief he looked upon her record with the greatest satisfaction.

Sir Thomas Goodwin, D.G.

**Sir Thomas Goodwin** incidentally referred to the Registration Bill for Nurses, and the great question of hours now occupying all minds. That while sternly opposing overwork, he felt that the medical and also the nursing profession must aim higher and be above the confining, narrowing influences which must come if two such great professions were compressed within a schedule of working hours. His remarks were received with great applause. The singing of the National Anthem concluded this very successful evening.

## Figures and Tables

**Figure f1:**